# Keratitis following leech therapy for periocular eczematous dermatitis: a case report

**DOI:** 10.1186/s12906-022-03613-1

**Published:** 2023-01-02

**Authors:** Dilek Özkaya

**Affiliations:** Department of Ophthalmology, Süleyman Demirel University Faculty of Medicine, 32260 Isparta, Turkey

**Keywords:** Medicinal leech therapy, Infection, Keratitis, Corneal scar, Corneal neovascularization

## Abstract

**Background:**

The medicinal leech therapy (MLT) is a kind of complementary treatment method used for various diseases. The leeches (*Hirudo medicinalis*) have been used for more than 2500 years by surgeons. The substances presenting in the saliva of leeches have anti-inflammatory, anticoagulant, platelet inhibitory, thrombin regulatory, analgesic, extracellular matrix degradative and antimicrobial effects. The method is cheap, easy to apply, effective and its mechanisms of action have been clarified for specific diseases. Infection particularly *Aeromonas* infection is the most common complication of MLT.

**Case presentation:**

In this case report, a keratitis case developing after leech therapy applied for the periocular and facial eczematous dermatitis lesions will be presented. The patient referred to our hospital with decreased vision, ocular pain, stinging, redness and lacrimation complaints. A large corneal epithelial defect with irregular margins, dying by fluorescein, involving more than inferior half of cornea and conjunctival hyperemia were seen in the right eye. No agent was determined in microbiological investigation, as the patient had used topical moxifloxacin eye drop which was commenced in another clinic before applying to us. The patient was treated with fortified vancomycin and ceftazidime, before using besifloxacin with the diagnosis of bacterial keratitis. Three weeks later epithelial defect improved completely leaving an opacity and neovascularization.

**Conclusions:**

MLT should be performed by certified physicians with sterile medicinal leeches and precautious antibiotics should be used before MLT for prevention against potential infections.

## Background

Medicinal leech therapy (MLT) or hirudotherapy is one of the traditional and complementary medicine methods. The discovery of hirudin, an anti-coagulant material, in leech saliva, increased the popularity of MLT in 1884 for the treatment of varicosis, venous insufficiency, and hemorrhoids. MLT regained importance in especially reconstructive surgery for providing blood-letting in the 1980s. Finally, Food and Drug Administration approved the MLT as a medical application in 2004 [1, 2].

Although more than 100 different bioactive molecules are presented in leech secretions, only some of them have been identified as possessing a main active role. These molecules have analgesic and anti-inflammatory, increasing blood flow, inhibition of platelet function, anticoagulant, and antimicrobial effects. The complications of MLT are infections, bleeding, anemia, and allergic reactions. *Aeromonas* species are the major pathogens leading to infections [3, 4].

The cornea is an avascular, transparent tissue acting as a barrier and protecting the eye. The nutrition of the cornea is supplied mainly by the aqueous humor posteriorly and the tears anteriorly. Also, the capillaries from anterior ciliary arteries form plexuses around the cornea. The cornea should be transparent for providing a suitable optical surface. Its transparency depends on avascularity, regular arrangement of collagen fibers, and stability of cellular elements and layers [5, 6]. The risk factors for keratitis include contact lens wear, ocular trauma, ocular surgery, and ocular surface disease [ 7]. In this case report, a patient developing microbial keratitis without a history of any risk factor, following leech therapy for periocular eczematous dermatitis will be presented.

## Case presentation

An 85 years old female patient was referred to our hospital with complaints of decreased vision, ocular pain, redness, and lacrimation in her right eye. Her complaints had begun 5 days after leech therapy applied for the facial and periocular eczematous dermatitis. While MLT should be performed by a certified physician with sterile medicinal leeches, the application had been performed by one of her relatives at home. She had no history of trauma or ocular surface disorder. She had presented to another hospital for her ocular complaints and moxifloxacin (Vigamox®, Alcon Laboratories, Fort Worth, TX) was commenced before coming to us. She had been referred to our hospital, as there was no improvement in her clinical signs. Institutional Review Board approval was not required for this case report, because according to the national guidelines, the article is exempt from ethical committee approval as it is a case report of a patient previously seen.

**Fig. 1:**
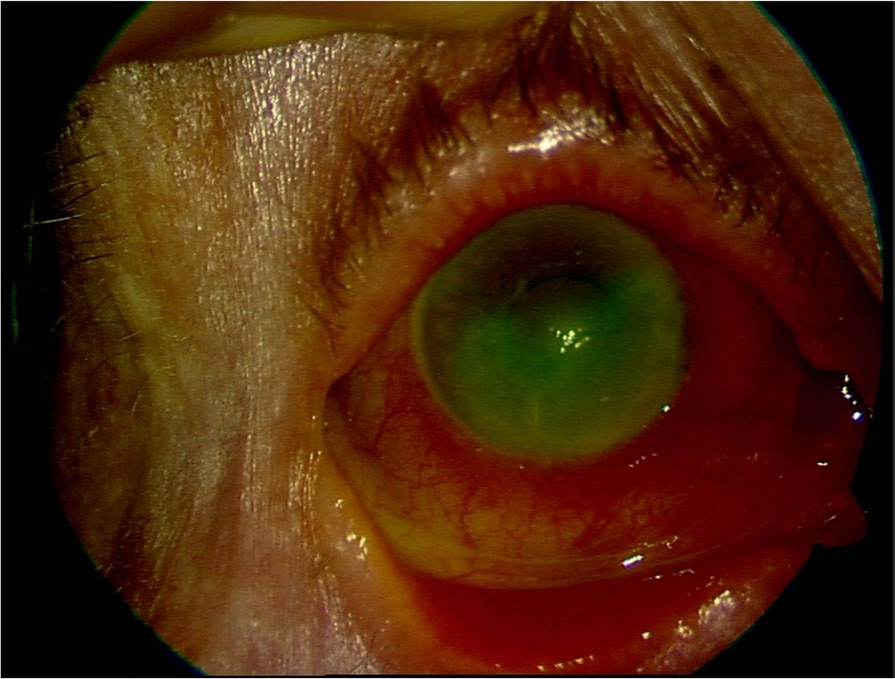
A large corneal epithelial defect, dying by fluorescein and involving more than the inferior half of the cornea, silier injection, and stromal infiltration (first examination, day 0)

**Fig. 2:**
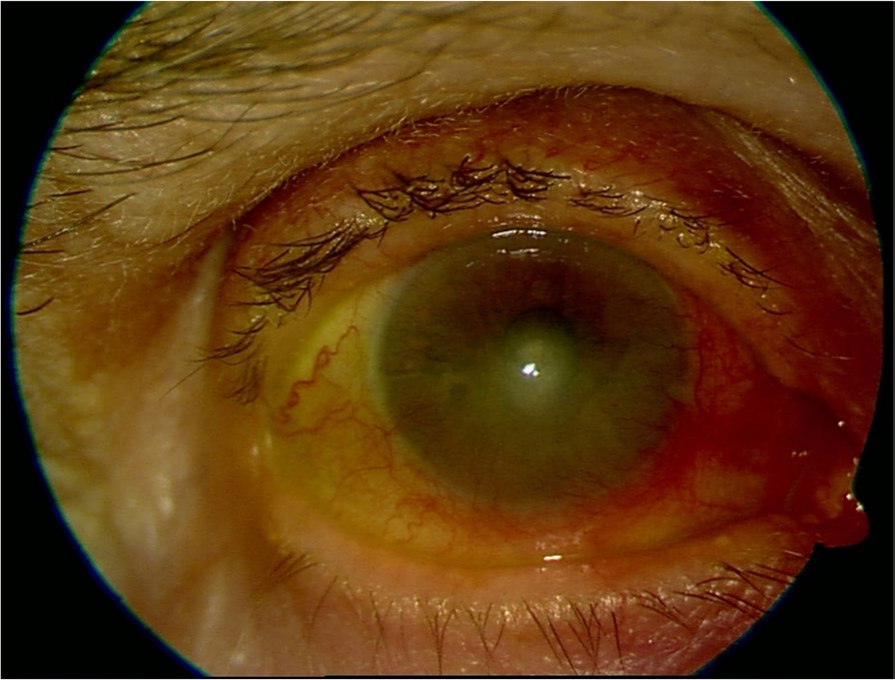
Neovascularization and opacity involving more than the inferior half of the cornea, the lesion not dyed by fluorescein, and regressed silier injection (day 30)

The patient’s best-corrected visual acuity (BCVA) was hand motion in the right, and 0.4 in the left eye. Slit-lamp examination revealed a large corneal epithelial defect sized 8 × 10 mm, with irregular margins, dying by fluorescein and involving more than the inferior half of the cornea in her right eye (shown in Fig. 1). Conjunctiva was also hyperemic in the right eye. The left eye’s biomicroscopic examination revealed a nuclear cataract. The patient was hospitalized with the diagnosis of infective keratitis and corneal scrapings were sent for microbiological investigation. Topical fortified vancomycin (Edicin®, Lek Pharmaceutical, Slovenia) and ceftazidime (Fortum®, GlaxoSmithKlein, Italy), cyclopentolate (Sikloplejin®, İdol Drug, İstanbul), cross-linked hyaluronic acid (Visu XL®, VISUfarma, Italy) without preservatives and dexpanthenol (Recugel®, Bausch Lomb, Canada) were commenced. The microbiological investigation did not identify any pathogen as she had been using topical moxifloxacin (Vigamox®, Alcon Laboratories, Fort Worth, TX) for 5 days before applying to us. The size of epithelial defect started to decrease slightly in 4-5 days. Topical loteprednol etabonate (Lotemax®, Bausch Lomb, Canada) was added following epithelial closure. After ceasing the fortified antibiotics, the patient was continued on 0.6% besifloxacin (Besivance®, Bausch & Lomb Incorporated, Tampa, Florida). Complete re-epithelization of the lesion took 3 weeks and the final BCVA was 0.05 in the right eye. Neovascularization and opacity involving more than the inferior half of the cornea were observed at the last examination (shown in Fig. 2).

## Discussion and conclusions

Infective keratitis is one of the most common causes of corneal blindness, especially in developing countries. Bacterial keratitis forms 50-60% of infective keratitis and *Staphylococcus*, *Streptococcus*, and *Pseudomonas* species are the most common pathogens. Ocular pain, decreased vision, and photophobia are the symptoms of bacterial keratitis. Clinical signs include ulceration of epithelium, anterior chamber reaction with or without hypopyon, and stromal infiltration with edema. Contact lens wear, ocular surface disorder, corneal trauma, and surgery are predisposing factors. Fortified topical antibiotics are recommended for large or vision-threatening corneal infiltrates and hypopyon [8].

MLT has been successfully used in plastic and reconstruction surgery to improve the revascularization of flaps, grafts, and replants. Additionally, osteoarthritis, cardiovascular diseases due to blood coagulation disorders, diabetic foot ulcers, migraine headache, dermatitis, macroglossia, and wounds are the other indications of MLT. Ophthalmic indications of MLT are reported as cataract, glaucoma, traumatic injuries, and intraocular inflammation. Infection is the most common complication of MLT. The rate of infection following MLT is reported as 2.4-20% in the literature [9, 10]. *Aeromonas*, *Pseudomonas,* and *Vibrio* species are the agents that cause infections due to MLT. The major infectious agent, *Aeromonas hydrophila,* leads to wound infection, local abscess, cellulitis, peritonitis, osteomyelitis, pneumonia, and sepsis. All isolated agents are sensitive to third-generation cephalosporins and ciprofloxacin. Therefore, antibiotic prophylaxis with fluoroquinolones such as ofloxacin 200 mg twice a day is recommended for preventing infective complications [11].

There are two case reports notifying the periocular infections due to MLT in the literature. Firstly, Gülyeşil et al. reported a periorbital cellulitis case after MLT for glaucoma treatment. The patient was admitted because of redness, pain, and swelling around her right eye. She was successfully treated with oral ciprofloxacin and flurbiprofen [12]. Secondly, Kılıç et al. reported an orbital cellulitis case after a leech therapy applied for headache. The patient presented with complaints of swelling and redness in both eyes and face. The diagnosis was orbital cellulitis due to leech therapy and the patient was hospitalized for treatment of orbital cellulitis [13]. A case of keratitis developing due to leech therapy could not be found in the literature. Therefore, this case report is the first one presenting a case of keratitis following leech therapy. The limitation of this case report is that any microbial growth could not be detected, as the patient had a history of topical antibiotic treatment.

In conclusion, MLT should be performed by a health professional, after the evaluation of the patient clinically by a physician. Prophylactic antibiotic treatment covering the most frequent isolated agents may enhance the safety of application by decreasing the rates of infections due to MLT.

## Data Availability

All data generated or analyzed during this study are included in this published article.

## Abbreviations

MLT: Medicinal leech therapy
BCVA: Best-corrected visual acuity (BCVA)
